# Integrated mendelian randomization analyses highlight AFF3 as a novel eQTL-mediated susceptibility gene in renal cancer and its potential mechanisms

**DOI:** 10.1186/s12885-024-12513-1

**Published:** 2024-06-17

**Authors:** Qiming Wang, Shaopeng Chen, Gang Wang, Tielong Zhang, Yulong Gao

**Affiliations:** Department of Urology, Jianhu Clinical Medical College of Yangzhou University, No. 666 South Ring Road, Yancheng, Jiangsu Province 224700 China

**Keywords:** Mendelian randomization, eQTL, SMR, AFF3, Renal cancer

## Abstract

**Backgrounds:**

A growing number of expression quantitative trait loci (eQTLs) have been found to be linked with tumorigenesis. In this article, we employed integrated Mendelian randomization (MR) analyses to identify novel susceptibility genes in renal cancer (RC) and reveal their potential mechanisms.

**Methods:**

Two-sample MR analyses were performed to infer causal relationships between eQTLs, metabolites, and RC risks through the “TwoSampleMR” R package. Sensitivity analyses, such as heterogeneity, pleiotropy, and leave-one-out analysis, were used to assess the stability of our outcomes. Summary-data-based MR (SMR) analyses were used to verify the causal relationships among cis-eQTLs and RC risks via the SMR 1.3.1 software.

**Results:**

Our results provided the first evidence for AFF3 eQTL elevating RC risks, suggesting its oncogenic roles (IVW method; odds ratio (OR) = 1.0005; 95% confidence interval (CI) = 1.0001–1.0010; *P* = 0.0285; heterogeneity = 0.9588; pleiotropy = 0.8397). Further SMR analysis validated the causal relationships among AFF3 cis-eQTLs and RC risks (*P* < 0.05). Moreover, the TCGA-KIRC, the ICGC-RC, and the GSE159115 datasets verified that the AFF3 gene was more highly expressed in RC tumors than normal control via scRNA-sequencing and bulk RNA-sequencing (*P* < 0.05). Gene set enrichment analysis (GSEA) analysis identified six potential biological pathways of AFF3 involved in RC. As for the potential mechanism of AFF3 in RC, we concluded in this article that AFF3 eQTL could negatively modulate the levels of the X-11,315 metabolite (IVW method; OR = 0.9127; 95% CI = 0.8530–0.9765; *P* = 0.0081; heterogeneity = 0.4150; pleiotropy = 0.8852), exhibiting preventive effects against RC risks (IVW method; OR = 0.9987; 95% CI = 0.9975–0.9999; *P* = 0.0380; heterogeneity = 0.5362; pleiotropy = 0.9808).

**Conclusions:**

We concluded that AFF3 could serve as a novel eQTL-mediated susceptibility gene in RC and reveal its potential mechanism of elevating RC risks via negatively regulating the X-11,315 metabolite levels.

**Supplementary Information:**

The online version contains supplementary material available at 10.1186/s12885-024-12513-1.

## Introduction

Renal cancer (RC) is a highly vascularized neoplasm in the urinary system. Its incidence and mortality have been steadily rising globally in 2020, with 431,288 new cases and 179,368 new deaths [1]. Currently, surgical resection is still the foremost therapy option for early-stage RC [2]. Based on reported data, the 5-year survival rate for patients with early-stage RC can be as high as 95%. However, many patients are diagnosed at later stages when this cancer has already metastasized, leading to poorer prognoses of less than 10% [3, 4]. Although various advancements have been made in characterizing the genetic landscape of RC, our understanding of the molecular mechanisms driving tumorigenesis and progression remains incomplete [5]. Recently, the identification of novel gene signatures or biomarkers associated with RC development and progression represents a promising avenue to improve early detection, prognosis, and personalized therapy for this disease [6, 7]. Hence, critical needs were required to uncover new genomic and transcriptomic markers in RC that can serve as diagnostic, prognostic, and predictive biomarkers to ultimately improve these patients’ survivals.

Due to the principle that the allocation of genetic variants occurs randomly during meiosis, Mendelian randomization (MR), as a powerful epidemiological technique, utilizes genetic variants as instrumental variables (IVs) to infer causal relationships between modifiable exposures and disease outcomes, generally free from biases that hamper observational studies [8, 9]. Expression quantitative trait loci (eQTLs) were crucial in cancer research for linking genetic variants to gene expression changes, aiding in identifying cancer susceptibility genes and understanding tumor biology [10, 11]. They integrated GWAS data with gene expression profiles to pinpoint risk alleles affecting relevant tissues [12]. Applications of MR with eQTLs have been widely used in various tumor and non-tumor diseases [13–15]. Meng et al. shed light on the fact that the PLK4 cis-eQTL genetic variant rs3811741 could confer hepatocellular carcinoma high risks [16]. Dominguez-Alonso et al. highlighted nine novel susceptibility genes (KANSL1, CRHR1, MAPT, MANBA, NKX2-2, MMP12, PTPRE, WNT3, and SRPK2) in autism spectrum disorders via eQTL colocalization analysis [17]. These insights were vital for developing targeted therapies and improving diagnostic strategies for tumors, highlighting the importance of eQTLs in uncovering the genetic mechanisms driving cancer. However, eQTLs were seldomly studied in RC. In this article, we also integrated MR analyses to identify novel susceptibility genes and reveal their potential mechanisms in RC, providing promising prognostic biomarkers or therapeutic targets.

## Materials and methods

### Study design

**Fig. 1:**
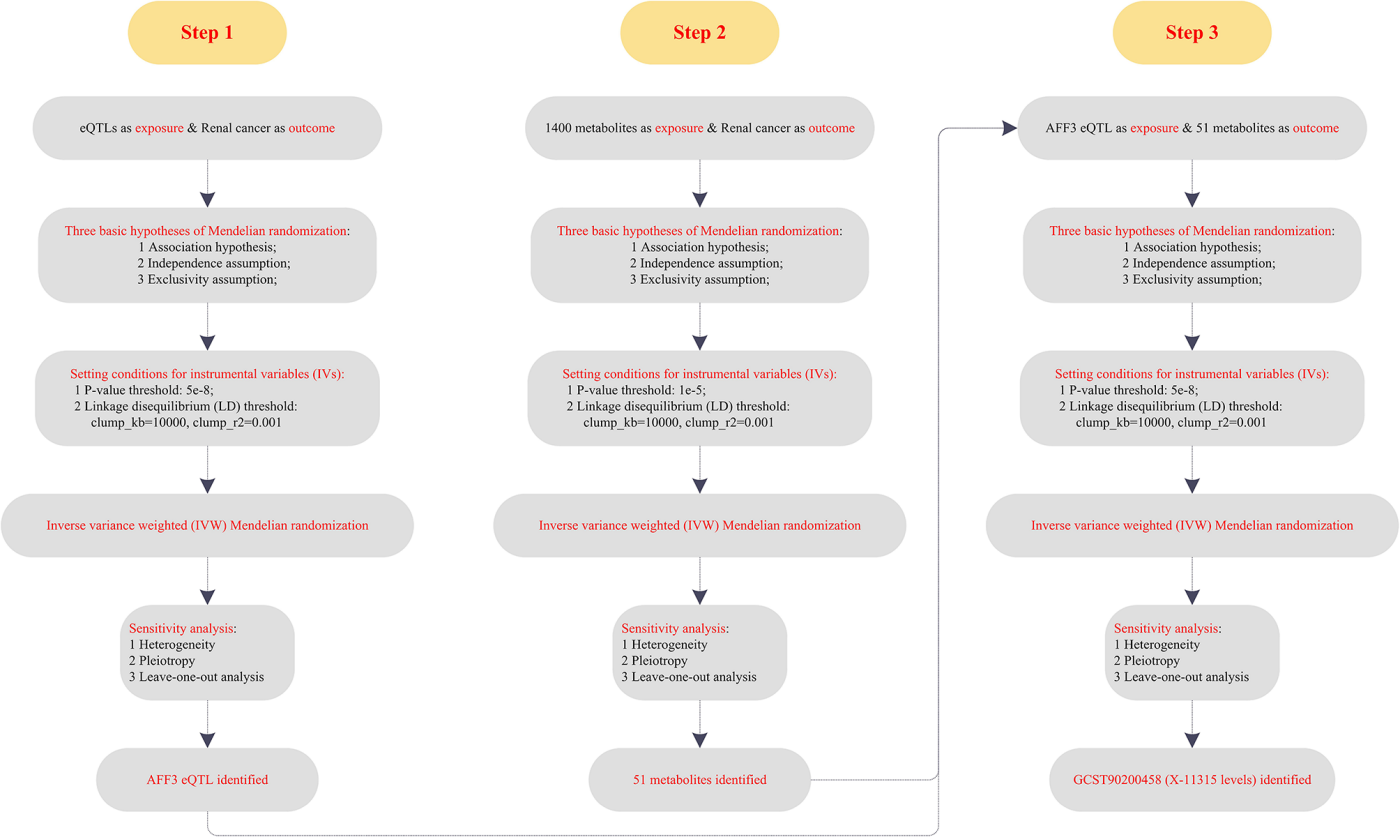
The whole study design

This article strictly adhered to the standards for Strengthening the Reporting of Observational Studies in Epidemiology (STROBE) checklist [18] and three basic MR assumptions [19]. The whole study design was detailed in Fig. 1, including three steps: Step 1 (19,942 gene eQTLs as exposure and RC as outcome); Step 2 (1,400 metabolites as exposure and RC as outcome); and Step 3 (AFF3 eQTL as exposure and 51 metabolites as outcome).

### Data sources

19,942 gene eQTLs genetic data were obtained from the online IEU OpenGWAS project website (https://gwas.mrcieu.ac.uk/), with GWAS IDs of eqtl-a-Ensembl IDs. Therein, the AFF3 eQTL genetic data included 26,609 samples and 17,880 single nucleotide polymorphisms (SNPs). 1,400 metabolite genetic data were obtained from the online GWAS Catalog website (https://www.ebi.ac.uk/gwas/), with the IDs GCST90199621 to GCST90201020 [20]. Therein, the X-11,315 metabolite with ID of GCST90200458 included 8,139 samples. RC genetic data was derived from the online IEU OpenGWAS project website (https://gwas.mrcieu.ac.uk/), with a GWAS ID of ukb-b-1316, containing 463,010 samples and 9,851,867 SNPs. The cis-eQTLs genetic data were obtained from the eQTLGen online website (https://www.eqtlgen.org/cis-eqtls.html). All the study populations involved in this article were European.

### Selection of IVs

If eQTLs were exposures, SNPs were selected as IVs when P value thresholds were below 5e-8; the default linkage disequilibrium (LD) thresholds were set at clumped kb = 10,000 as well as r2 = 0.001; and F-statistic thresholds were above 10 [21]. If RC or metabolites were exposures, SNPs were selected as IVs when P value thresholds were below 1e-5; the default linkage disequilibrium (LD) thresholds were set at clumped kb = 10,000 as well as r2 = 0.001; and F-statistic thresholds were above 10. All these selection criteria ensured the MR correlation assumption. Additionally, phenotype scanning (http://www.phenoscanner.medschl.cam.ac.uk/) was used to lessen the impact of confounding variables and ensure the MR independence assumption [22].

### MR analysis and sensitivity analyses

Two-sample MR analyses were applied in this article to infer causal relationships between modifiable exposures and disease outcomes through the “TwoSampleMR” R package [23]. During the analysis, the inverse variance weighted (IVW) approach was deemed the main outcome of this study, compared with the others (the MR Egger method, the weighted median method, the weighted mode method, and the simple mode method) [24]. P values below 0.05 were set as cut-off values and deemed to be statistically significant. In addition, we also applied sensitivity analyses in this study, such as heterogeneity, pleiotropy, and leave-one-out analysis, to assess the stability of our outcomes [25, 26]. Sensitivity analyses ensured the MR exclusivity assumption.

### Summary-data-based MR (SMR) analyses, single-cell RNA-sequencing (scRNA-seq) and bulk RNA-seq analyses

SMR analyses were used to verify the causal relationships among cis-eQTLs and RC risks via the SMR 1.3.1 software with default settings and cut-off values of *P* < 0.05 in the eQTLGen dataset. The heterogeneity in dependent instruments (HEIDI) test was used for sensitivity analysis [27]. Bulk RNA-seq data from the TCGA-KIRC and the ICGC-RC datasets were utilized to verify the AFF3 gene expression levels in RC tissues compared with normal controls, with the help of the R “limma” package and cut-off values of *P* < 0.05. ScRNA-seq data of RC were obtained from the GSE159115 dataset (https://www.ncbi.nlm.nih.gov/geo/query/acc.cgi?acc=GSE159115) [28], and it was used to verify the AFF3 gene single-cell expression levels in RC tissues compared with normal controls, with the help of the R “seurat” package and cut-off values of *P* < 0.05.

### Gene set enrichment analysis (GSEA)

Based on the median expression of AFF3 in the TCGA-KIRC datasets, the AFF3-high and AFF3-low subgroups were identified. GSEA analysis was conducted to seek AFF3-related pathways in these two groups with the help of the GSEA 4.0.0 software and the gene set of “c2.cp.kegg.v7.1.symbols.gmt” downloaded from the MSigDB website (https://www.gsea-msigdb.org/gsea/msigdb/). The absolute values of the normalized enrichment score (NES) above 1.5 and the nominal p-value below 0.05 were set as cut-off values.

### Statistical analysis

The “TwoSampleMR” R package as well as the R 4.2.1 version software (http://www.Rproject.org) were applied in this article to evaluate the causal relationships between modifiable exposures and disease outcomes. Besides, P values below 0.05 were deemed to be statistically significant.

## Results

### MR analysis identified AFF3 as a novel susceptibility gene in RC

**Fig. 2:**
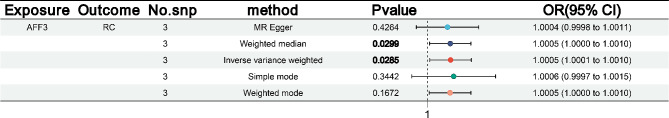
MR analysis revealed that genetic susceptibility to AFF3 eQTL could increase the risks of RC

The whole study design was detailed in Fig. 1, including three steps: Step 1 (19,942 gene eQTLs as exposure and RC as outcome); Step 2 (1,400 metabolites as exposure and RC as outcome); and Step 3 (AFF3 eQTL as exposure and 51 metabolites as outcome). Based on the results of step 1, we found that genetic susceptibility to AFF3 eQTL was able to increase the risks of RC (IVW method; odds ratio (OR) = 1.0005; 95% confidence interval (CI) = 1.0001–1.0010; *P* = 0.0285; heterogeneity = 0.9588; pleiotropy = 0.8397; Fig. 2). Its detailed forest plot, scatter plot, and leave-one-out analysis were presented in Figure S1. Taken together, MR analysis identified AFF3 as a novel susceptibility gene in RC.

### SMR analysis verified AFF3 as a novel susceptibility gene in RC

**Fig. 3:**
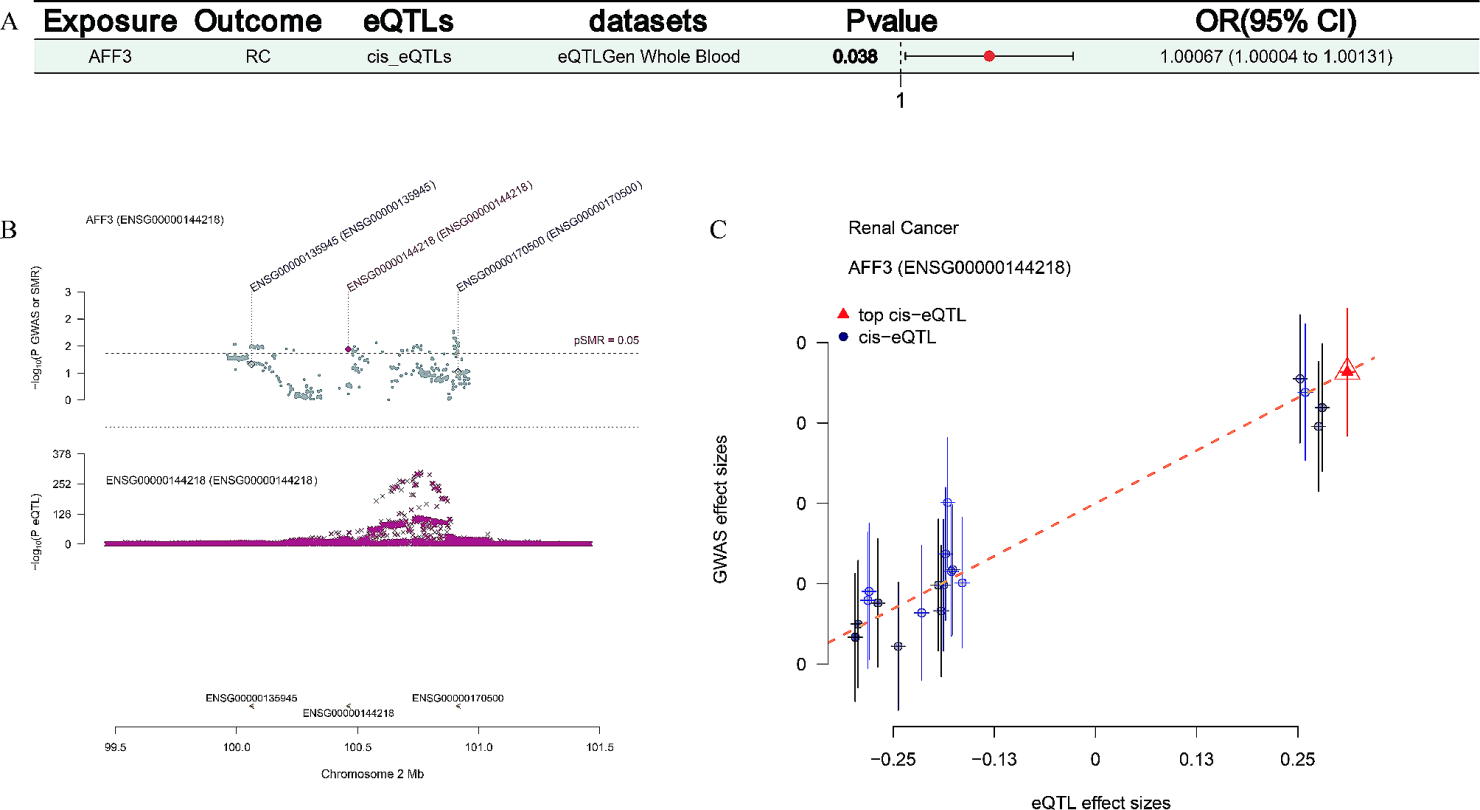
SMR analysis verified that genetic susceptibility to AFF3 cis-eQTL could increase the risks of RC; (**A**) The SMR forest plot; (**B**) The SMR locus plot; (**C**) The SMR correlation plot

SMR analyses were used to verify the causal relationships among AFF3 cis-eQTL and RC risks via the SMR 1.3.1 software with default settings and cut-off values of *P* < 0.05 in the eQTLGen dataset. Our results showed that genetic susceptibility to AFF3 cis-eQTL was also able to increase the risks of RC (OR = 1.00067; 95% CI = 1.00004-1.00131; *P* = 0.038; HEIDI = 0.992; Fig. 3A). The SMR locus plot was detailed in Fig. 3B. The SMR correlation plot presented that they had a positive correlation among the AFF3 cis-eQTL and the RC GWAS data (Fig. 3C).

### ScRNA-seq and bulk RNA-seq analyses validated the AFF3 expressions in RC

**Fig. 4:**
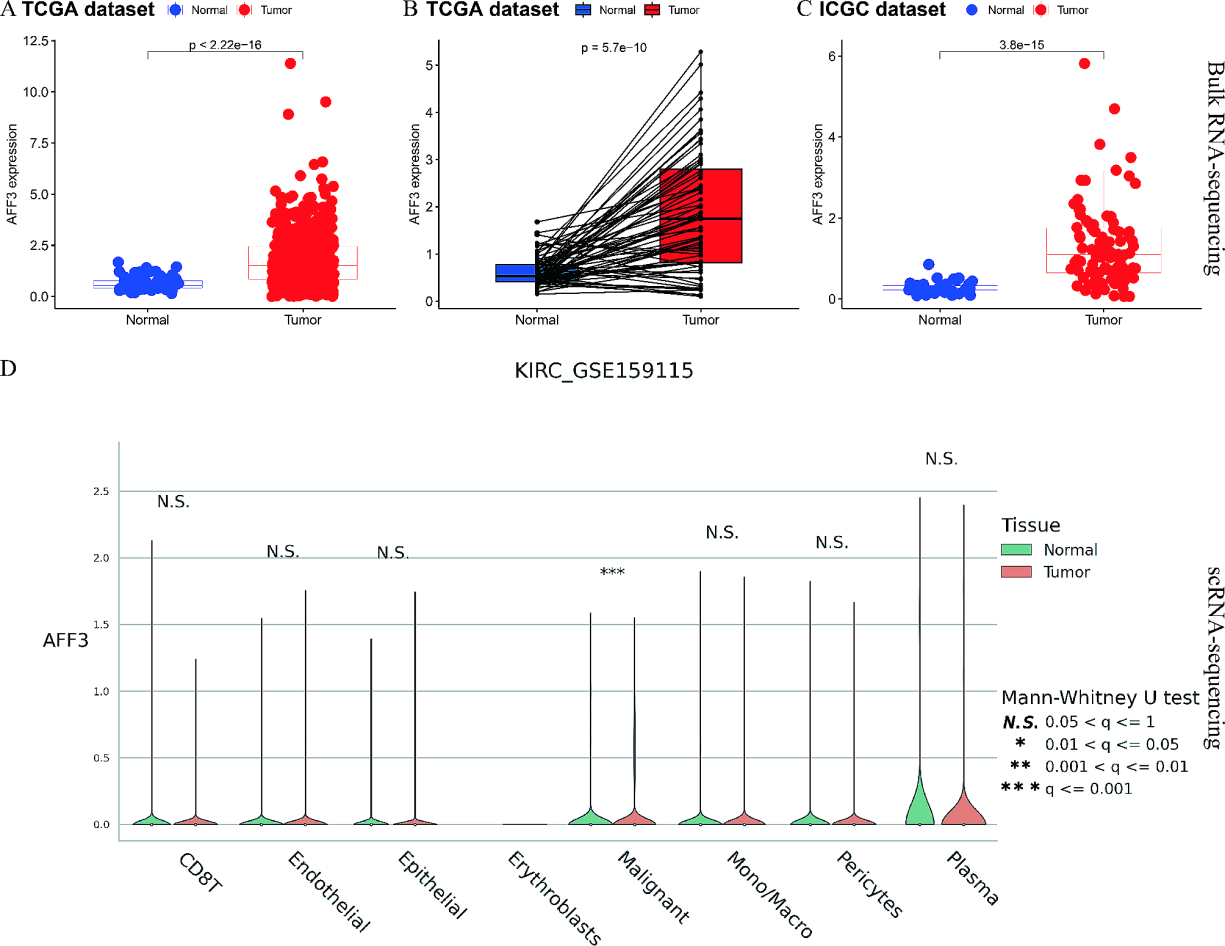
ScRNA-seq and bulk RNA-seq analyses validated the AFF3 expressions in RC; (**A**) the whole TCGA-KIRC bulk RNA-seq dataset (*N* = 72; T = 539); (**B**) the paired TCGA-KIRC bulk RNA-seq dataset (*N* = 72; T = 72); (**C**) the ICGC-RC bulk RNA-seq dataset (*N* = 45; T = 91); (**C**) the GSE159115 scRNA-seq dataset; *** means *P* < 0.05

Bulk RNA-seq data from the TCGA-KIRC and the ICGC-RC datasets were utilized to verify the AFF3 gene expression levels in RC tissues compared with normal controls, with the help of the R “limma” package and cut-off values of *P* < 0.05. Our results showed that the AFF3 gene had higher expression levels in RC tumors than normal control, in the whole TCGA-KIRC dataset (*N* = 72; T = 539; *P* < 0.001; Fig. 4A), in the paired TCGA-KIRC dataset (*N* = 72; T = 72; *P* < 0.001; Fig. 4B), and in the ICGC-RC dataset (*N* = 45; T = 91; *P* < 0.001; Fig. 4C). ScRNA-seq data from the GSE159115 dataset was used to verify the AFF3 gene single-cell expression levels in RC tissues compared with normal controls, with the help of the R “seurat” package and cut-off values of *P* < 0.05. Our results also presented that the AFF3 gene was more highly expressed in RC tumor cells than in normal cells (*P* < 0.001; Fig. 4D). Taken together, the AFF3 had a higher expression in RC tumors than normal control via scRNA-seq and bulk RNA-seq analyses.

### GSEA analysis identified AFF3-related pathways in RC

**Fig. 5:**
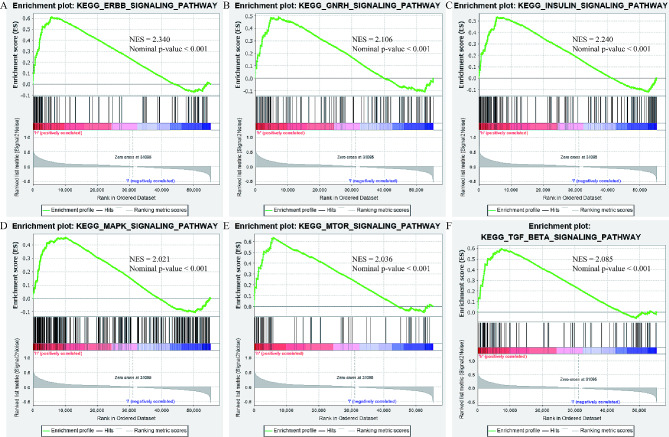
GSEA analysis identified AFF3-related pathways in RC; (**A**) the ERBB; (**B**) the GNRH; (**C**) the Insulin; (**D**) the MAPK; (**E**) the MTOR; and (**F**) the TGF beta pathways; NES: normalized enrichment score;

With the help of the GSEA 4.0.0 software and the gene set of “c2.cp.kegg.v7.1.symbols.gmt”, GSEA analysis was conducted to seek AFF3-related pathways in the AFF3-high and AFF3-low subgroups based on the median expression. As detailed in Fig. 5, we found that AFF3 was markedly linked with the ERBB, the GNRH, the Insulin, the MAPK, the MTOR, and the TGF beta pathways (all the absolute values of NES were above 1.5 and the nominal p-values were below 0.05). All of these indicated the potential biological pathways of AFF3 involved in RC.

### MR analysis revealed the potential mechanism of AFF3 in RC

**Fig. 6:**
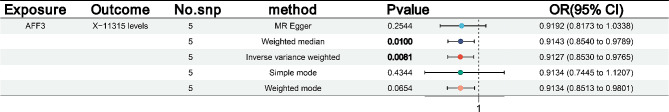
MR analysis revealed that genetic susceptibility to AFF3 eQTL could decrease the risks of the X-11,315 metabolite levels

**Fig. 7:**
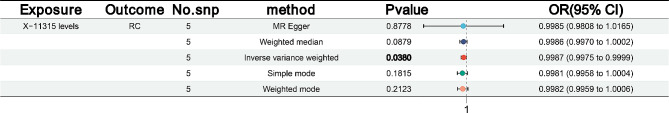
MR analysis revealed that genetic susceptibility to the X-11,315 metabolite levels could decrease the risks of RC

**Fig. 8:**
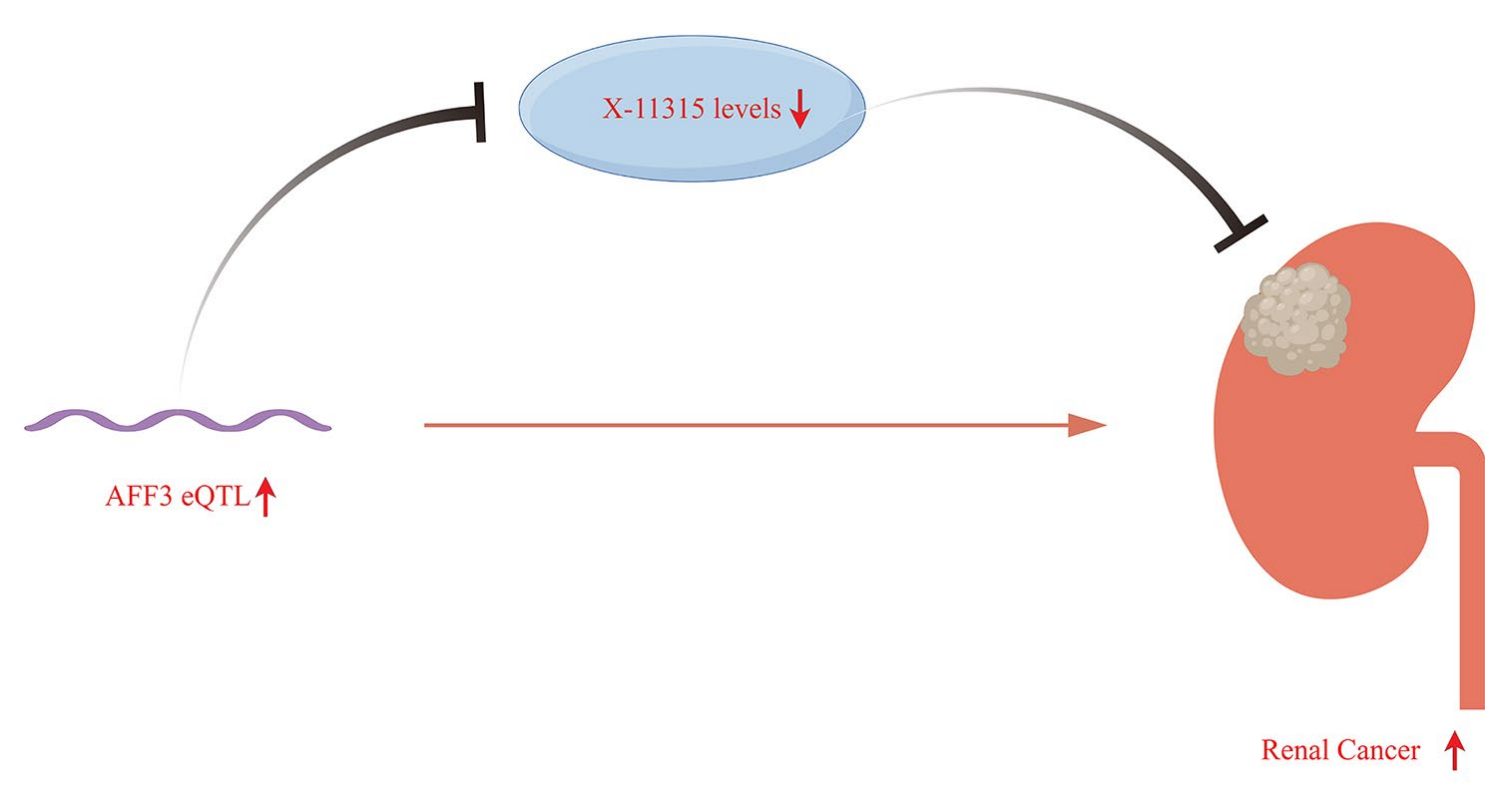
Sketch map of the potential mechanism of AFF3 in RC via regulating the X-11,315 metabolite levels

To reveal the potential mechanism of AFF3 in RC, steps 2 and 3 were further conducted. Based on their results, the X-11,315 metabolite levels were finally identified. We found that genetic susceptibility to AFF3 eQTL could decrease the risks of the X-11,315 metabolite levels (IVW method; OR = 0.9127; 95% CI = 0.8530–0.9765; *P* = 0.0081; heterogeneity = 0.4150; pleiotropy = 0.8852; Fig. 6). Its detailed forest plot, scatter plot, and leave-one-out analysis were presented in Figure S2. Moreover, genetic susceptibility to the X-11,315 metabolite levels could also decrease the risks of RC (IVW method; OR = 0.9987; 95% CI = 0.9975–0.9999; *P* = 0.0380; heterogeneity = 0.5362; pleiotropy = 0.9808; Fig. 7). Its detailed forest plot, scatter plot, and leave-one-out analysis were presented in Figure S3. Taken together, we concluded that AFF3 eQTL increased the risks of RC via regulating the X-11,315 metabolite levels and its sketch map was detailed in Fig. 8.

## Discussion

RC represents one of the most prevalent malignancies of the urinary system, with its incidence and mortality continuously rising globally, which severely compromises patients’ prognosis [29]. Identifying novel pathogenic mechanisms and therapeutic targets shows great promise to improve early diagnosis and precision treatment for RC [30, 31]. MR analysis, as an instrumental variable approach, determines causality between exposures and outcomes by leveraging the natural random assortment of alleles during conception [32]. Hence, in this article, we applied MR analysis associated with eQTL analysis to identify novel susceptibility genes and reveal their potential mechanisms in RC, providing promising prognostic biomarkers or therapeutic targets for these patients.

Based on our study design, a total of three steps were conducted sequentially, including Step 1 (19,942 gene eQTLs as exposure and RC as outcome); Step 2 (1,400 metabolites as exposure and RC as outcome); and Step 3 (AFF3 eQTL as exposure and 51 metabolites as outcome). Our results showed that genetic susceptibility to AFF3 eQTL was able to increase the risks of RC, indicating AFF3 as a novel susceptibility gene in RC. Further SMR analysis validated the causal relationships among AFF3 cis-eQTLs and RC risks. Moreover, the TCGA-KIRC, the ICGC-RC, and the GSE159115 datasets verified that the AFF3 gene was more highly expressed in RC tumors than normal control via scRNA-seq and bulk RNA-seq. GSEA analysis identified six potential biological pathways of AFF3 involved in RC. To further reveal the potential mechanism of AFF3 in RC, steps 2 and 3 were conducted, and the X-11,315 metabolite levels were identified. Our results finally concluded that AFF3 eQTL could increase the risks of RC through regulating the X-11,315 metabolite levels. This logical workflow not only discovered multiple novel genes and metabolites associated with RC risk, but also informed future functional analyses to focus on these positive findings, thereby improving research efficiency. More importantly, it laid the groundwork for elucidating the molecular mechanisms connecting gene expression, metabolic activities, and cancer development. Further in-depth investigations were warranted based on the clues uncovered here.

ALF transcription elongation factor 3 (AFF3), also known as MLLT2-like, LAF4, and KINS, belonged to the AF4/FMR2 family and encoded a nuclear protein involving multiple biological processes, including autoimmune diseases, tumorigenesis, intellectual disability, horseshoe kidney, and so on [33–35]. Zeng et al. found that AFF3 could serve as a new therapeutic target for gastric cancer immunotherapy [35]. Cen et al. revealed that the significant associations among AFF3 rs10865035 polymorphisms and systemic lupus erythematosus in Chinese [36]. Shi et al. shed light on the fact that upregulation of AFF3 could mediate the resistance of tamoxifen in breast cancer [37]. However, little was currently known about the roles of AFF3 in RC. Our study provided the first evidence for AFF3 eQTL elevating RC risks, suggesting its oncogenic roles. Further SMR analysis validated the causal relationships among AFF3 cis-eQTLs and RC risks. Further biological investigations are required to depict how AFF3 mechanistically contributed to RC onset and development. More efforts were also warranted to assess the potential of AFF3 as a biomarker or therapeutic target for the precision diagnosis and treatment of RC patients.

To reveal the potential biological pathways of AFF3 involved in RC, GSEA analysis was conducted, and the ERBB, the GNRH, the Insulin, the MAPK, the MTOR, and the TGF beta pathways were identified. Most of these pathways had been reported to play vital roles in RC. Yao et al. reported that the ERBB and MAPK pathways were significantly enriched in RC via bioinformatics analysis in 2016 [38]. Solarek et al. showed that insulin and insulin-like growth factors could serve as the intratumoral regulators of RC [39]. Roldán-Romero et al. revealed that the loss of the deubiquitinase gene USP9X could sensitize RC cells to mTOR inhibition [40]. Nam et al. shed light on that the TGF-β/HDAC7 pathway would suppress the metabolism of the TCA cycle in RC [41]. Taken together, our results revealed the potential biological pathways of AFF3 involved in RC, which deserved to be further explored.

A growing number of metabolites had been found to act as critical effector molecules to influence various malignant properties and serve as important biomarkers reflecting tumor metabolic phenotypes for cancer detection and surveillance [42, 43]. Boyland et al. revealed that the metabolites of 7,12-dimethylbenz(a)anthracene could induce adrenal damage and tumorigenesis [44]. Yang et al. summarized the correlations among gut microbiota-derived metabolites and cancer progression or therapy [45]. Nagel et al. investigated the associations between metabolic factors and small intestine cancer risks, showing that interacted metabolic factors and elevated triglycerides would lead to increased small intestine cancer risks in women [46]. In this article, our study revealed the preventative effects of X-11,315 against RC, implying the metabolite’s modulation as a potential anti-RC strategy. As reported by Talmor-Barkan et al., they discovered a Clostridiaceae family bacterium species that was previously unidentified, indexing SGB 4712. X-11,315 was thought to originate from diet and was positively linked with SGB 4712 [47]. Other information regarding the properties and functions of X-11,315 remained largely unknown. Further deciphering the connections between gene regulation, metabolic reprogramming, and RC pathogenesis would pave the way for these patients’ novel diagnostic and therapeutic developments.

As for the potential mechanism of AFF3 in RC, we concluded in this article that AFF3 eQTL could negatively modulate the levels of the X-11,315 metabolite, exhibiting preventive effects against RC risks. Although the exact identity and functions of X-11,315 remain unclear at the current stage, this finding undoubtedly provides clues guiding future developments of metabolic biomarker-based diagnostic tests and therapies for RC. Similar mechanisms could be found in other diseases, such as asthma [48], multiple sclerosis [49], and so on. Deeper investigations into the interplay between AFF3 and metabolic networks could yield more fruitful outcomes moving forward.

Certain limitations also existed in our study. Firstly, since MR analysis was merely a technique used to examine the causal links between exposures and outcomes, it cannot be utilized as a substitute for clinical trials in the objective world [50]. Further studies or experiments were needed to validate AFF3’s role in RC. Secondly, baseline characteristics among controls and cases remained unclear, which might introduce residual confounding and cannot further stratify the study population [23]. Finally, all the study populations involved in this article were European, which was linked to sample selection bias [24]. The results in other populations should be interpreted with caution. Future studies were needed to conduct in-depth experiments based on the current study results to further explore the role of AFF3 in RC and possible intervention strategies.

## Conclusions

In this article, our study provided the first evidence for AFF3 eQTL elevating RC risks, suggesting its oncogenic roles. Further SMR analysis validated the causal relationships among AFF3 cis-eQTLs and RC risks. Moreover, the TCGA-KIRC, the ICGC-RC, and the GSE159115 datasets verified that the AFF3 gene was more highly expressed in RC tumors than normal control via scRNA-seq and bulk RNA-seq. GSEA analysis identified six potential biological pathways of AFF3 involved in RC. As for the potential mechanism of AFF3 in RC, we concluded in this article that AFF3 eQTL could negatively modulate the levels of the X-11,315 metabolite, exhibiting preventive effects against RC risks. The outcomes of us laid the groundwork for elucidating the molecular mechanisms connecting gene expression, metabolic activities, and cancer development. Further in-depth investigations were warranted based on the clues uncovered here.

### Electronic supplementary material

Below is the link to the electronic supplementary material.

Supplementary Material 1

Supplementary Material 2

Supplementary Material 3

Supplementary Material 4

Supplementary Material 5

## Data Availability

eQTLs or RC genetic data were obtained from the online IEU OpenGWAS project website (https://gwas.mrcieu.ac.uk/), with GWAS IDs of eqtl-a-Ensembl IDs or ukb-b-1316. 1,400 metabolite genetic data were obtained from the online GWAS Catalog website (https://www.ebi.ac.uk/gwas/), with the IDs GCST90199621 to GCST90201020. The cis-eQTLs genetic data were obtained from the eQTLGen online website (https://www.eqtlgen.org/cis-eqtls.html). ScRNA-sequencing and bulk RNA-sequencing data were obtained from the TCGA-KIRC, the ICGC-RC, and the GSE159115 datasets.

## References

[1] Sung H, Ferlay J, Siegel RL, Laversanne M, Soerjomataram I, Jemal A, Bray F (2021). Global Cancer statistics 2020: GLOBOCAN estimates of incidence and Mortality Worldwide for 36 cancers in 185 countries. Cancer J Clin.

[2] Song H, Xu B, Luo C, Zhang Z, Ma B, Jin J, Zhang Q (2019). The prognostic value of preoperative controlling nutritional status score in non-metastatic renal cell carcinoma treated with surgery: a retrospective single-institution study. Cancer Manage Res.

[3] Chaffer CL, Weinberg RA (2011). A perspective on cancer cell metastasis. Sci (New York NY).

[4] Hirata H, Hinoda Y, Ueno K, Nakajima K, Ishii N, Dahiya R (2012). MicroRNA-1826 directly targets beta-catenin (CTNNB1) and MEK1 (MAP2K1) in VHL-inactivated renal cancer. Carcinogenesis.

[5] Zhang X, Bolck HA, Rupp NJ, Moch H. Genomic alterations and diagnosis of renal cancer. Virchows Archiv: Int J Pathol 2023. 10.1007/s00428-023-03700-9 PMC10948545 37999735

[6] Oto J, Plana E, Sánchez-González JV, García-Olaverri J, Fernández-Pardo Á, España F, Martínez-Sarmiento M, Vera-Donoso CD, Navarro S, Medina P (2020). Urinary microRNAs: looking for a New Tool in diagnosis, prognosis, and monitoring of Renal Cancer. Curr Urol Rep.

[7] Wistuba-Hamprecht K, Gouttefangeas C, Weide B, Pawelec G (2020). Immune signatures and Survival of patients with metastatic melanoma, Renal Cancer, and breast Cancer. Front Immunol.

[8] Dobrijevic E, van Zwieten A, Kiryluk K, Grant AJ, Wong G, Teixeira-Pinto A (2023). Mendelian randomization for nephrologists. Kidney Int.

[9] Larsson SC, Butterworth AS, Burgess S (2023). Mendelian randomization for cardiovascular diseases: principles and applications. Eur Heart J.

[10] Liu Y, Gusev A, Kraft P (2023). Germline Cancer gene expression quantitative trait loci are Associated with local and global tumor mutations. Cancer Res.

[11] Zhu Y, Peng X, Wang X, Ying P, Wang H, Li B, Li Y, Zhang M, Cai Y, Lu Z (2022). Systematic analysis on expression quantitative trait loci identifies a novel regulatory variant in ring finger and WD repeat domain 3 associated with prognosis of pancreatic cancer. Chin Med J.

[12] Brown R, Kichaev G, Mancuso N, Boocock J, Pasaniuc B (2017). Enhanced methods to detect haplotypic effects on gene expression. Bioinf (Oxford England).

[13] Nguyen JP, Arthur TD, Fujita K, Salgado BM, Donovan MKR, Matsui H, Kim JH, D’Antonio-Chronowska A, D’Antonio M, Frazer KA (2023). eQTL mapping in fetal-like pancreatic progenitor cells reveals early developmental insights into diabetes risk. Nat Commun.

[14] Wang X, Gharahkhani P, Levine DM, Fitzgerald RC, Gockel I, Corley DA, Risch HA, Bernstein L, Chow WH, Onstad L (2022). eQTL Set-Based Association Analysis Identifies Novel Susceptibility Loci for Barrett Esophagus and Esophageal Adenocarcinoma. *Cancer epidemiology, biomarkers & prevention: a publication of the American Association for Cancer Research*. Cosponsored Am Soc Prev Oncol.

[15] Yoo T, Joo SK, Kim HJ, Kim HY, Sim H, Lee J, Kim HH, Jung S, Lee Y, Jamialahmadi O (2021). Disease-specific eQTL screening reveals an anti-fibrotic effect of AGXT2 in non-alcoholic fatty liver disease. J Hepatol.

[16] Meng L, Zhou Y, Ju S, Han J, Song C, Kong J, Wu Y, Lu S, Xu J, Yuan W (2019). A cis-eQTL genetic variant in PLK4 confers high risk of hepatocellular carcinoma. Cancer Med.

[17] Dominguez-Alonso S, Carracedo A, Rodriguez-Fontenla C (2023). eQTL colocalization analysis highlights novel susceptibility genes in Autism Spectrum disorders (ASD). Translational Psychiatry.

[18] Skrivankova VW, Richmond RC, Woolf BAR, Davies NM, Swanson SA, VanderWeele TJ, Timpson NJ, Higgins JPT, Dimou N, Langenberg C (2021). Strengthening the reporting of observational studies in epidemiology using mendelian randomisation (STROBE-MR): explanation and elaboration. BMJ (Clinical Res ed).

[19] Sanderson E, Glymour MM, Holmes MV, Kang H, Morrison J, Munafò MR, Palmer T, Schooling CM, Wallace C, Zhao Q et al. Mendelian randomization. Nat Reviews Methods Primers 2022, 2. 10.1038/s43586-021-00092-5 PMC7614635 37325194

[20] Chen Y, Lu T, Pettersson-Kymmer U, Stewart ID, Butler-Laporte G, Nakanishi T, Cerani A, Liang KYH, Yoshiji S, Willett JDS (2023). Genomic atlas of the plasma metabolome prioritizes metabolites implicated in human diseases. Nat Genet.

[21] Chen L, Yang H, Li H, He C, Yang L, Lv G (2022). Insights into modifiable risk factors of cholelithiasis: a mendelian randomization study. Hepatology (Baltimore MD).

[22] Lin J, Zhou J, Xu Y (2023). Potential drug targets for multiple sclerosis identified through mendelian randomization analysis. Brain.

[23] Wang C, Zhu D, Zhang D, Zuo X, Yao L, Liu T, Ge X, He C, Zhou Y, Shen Z (2023). Causal role of immune cells in schizophrenia: mendelian randomization (MR) study. BMC Psychiatry.

[24] Zhang Y, Peng R, Chen Z, Zhang W, Liu Z, Xu S, Zhu H, Chen J, Zheng B (2023). Evidence for a causal effect of major depressive disorder, anxiety on prostatitis risk: a univariate and multivariate mendelian randomization study. Prostate.

[25] Hemani G, Bowden J, Davey Smith G (2018). Evaluating the potential role of pleiotropy in mendelian randomization studies. Hum Mol Genet.

[26] Burgess S, Bowden J, Fall T, Ingelsson E, Thompson SG (2017). Sensitivity analyses for robust causal inference from mendelian randomization analyses with multiple genetic variants. Epidemiol (Cambridge Mass).

[27] Krishnamoorthy S, Li GH, Cheung CL (2023). Transcriptome-wide summary data-based mendelian randomization analysis reveals 38 novel genes associated with severe COVID-19. J Med Virol.

[28] Zhang Y, Narayanan SP, Mannan R, Raskind G, Wang X, Vats P, Su F, Hosseini N, Cao X, Kumar-Sinha C et al. Single-cell analyses of renal cell cancers reveal insights into tumor microenvironment, cell of origin, and therapy response. *Proceedings of the National Academy of Sciences of the United States of America* 2021, 118(24). 10.1073/pnas.2103240118 PMC8214680 34099557

[29] Usher-Smith J, Simmons RK, Rossi SH, Stewart GD (2020). Current evidence on screening for renal cancer. Nat Reviews Urol.

[30] Ivanova E, Fayzullin A, Grinin V, Ermilov D, Arutyunyan A, Timashev P, Shekhter A. Empowering Renal Cancer Management with AI and Digital Pathology: Pathology, Diagnostics and Prognosis. Biomedicines 2023, 11(11). 10.3390/biomedicines11112875 PMC10669631 38001875

[31] Popławski P, Bogusławska J, Hanusek K, Piekiełko-Witkowska A. Nucleolar Proteins and non-coding RNAs: roles in Renal Cancer. Int J Mol Sci 2021, 22(23). 10.3390/ijms222313126 PMC8658237 34884928

[32] Fang A, Zhao Y, Yang P, Zhang X, Giovannucci EL. Vitamin D and human health: evidence from mendelian randomization studies. Eur J Epidemiol 2024. 10.1007/s10654-023-01075-4 38214845

[33] Tsukumo SI, Subramani PG, Seija N, Tabata M, Maekawa Y, Mori Y, Ishifune C, Itoh Y, Ota M, Fujio K (2022). AFF3, a susceptibility factor for autoimmune diseases, is a molecular facilitator of immunoglobulin class switch recombination. Sci Adv.

[34] Voisin N, Schnur RE, Douzgou S, Hiatt SM, Rustad CF, Brown NJ, Earl DL, Keren B, Levchenko O, Geuer S (2021). Variants in the degron of AFF3 are associated with intellectual disability, mesomelic dysplasia, horseshoe kidney, and epileptic encephalopathy. Am J Hum Genet.

[35] Zeng Y, Zhang X, Li F, Wang Y, Wei M (2022). AFF3 is a novel prognostic biomarker and a potential target for immunotherapy in gastric cancer. J Clin Lab Anal.

[36] Cen H, Leng RX, Wang W, Zhou M, Feng CC, Chen GM, Li R, Pan HF, Li XP, Ye DQ (2012). Association of AFF1 rs340630 and AFF3 rs10865035 polymorphisms with systemic lupus erythematosus in a Chinese population. Immunogenetics.

[37] Shi Y, Zhao Y, Zhang Y, AiErken N, Shao N, Ye R, Lin Y, Wang S (2018). AFF3 upregulation mediates tamoxifen resistance in breast cancers. J Experimental Clin cancer Research: CR.

[38] Yao T, Wang Q, Zhang W, Bian A, Zhang J (2016). Identification of genes associated with renal cell carcinoma using gene expression profiling analysis. Oncol Lett.

[39] Solarek W, Koper M, Lewicki S, Szczylik C, Czarnecka AM (2019). Insulin and insulin-like growth factors act as renal cell cancer intratumoral regulators. J cell Communication Signal.

[40] Roldán-Romero JM, Valdivia C, Santos M, Lanillos J, Maroto P, Anguera G, Calsina B, Martinez-Montes A, Monteagudo M, Mellid S (2023). Deubiquitinase USP9X loss sensitizes renal cancer cells to mTOR inhibition. Int J Cancer.

[41] Nam H, Kundu A, Karki S, Brinkley GJ, Chandrashekar DS, Kirkman RL, Liu J, Liberti MV, Locasale JW, Mitchell T et al. The TGF-β/HDAC7 axis suppresses TCA cycle metabolism in renal cancer. JCI Insight 2021, 6(22). 10.1172/jci.insight.148438 PMC8663777 34609963

[42] Das UN (1999). Essential fatty acids and their metabolites and cancer. Nutr (Burbank Los Angeles Cty Calif).

[43] Hecht SS (2002). Human urinary carcinogen metabolites: biomarkers for investigating tobacco and cancer. Carcinogenesis.

[44] Boyland E, Sims P, Huggins C (1965). Induction of adrenal damage and cancer with metabolites of 7,12-dimethylbenz(a)anthracene. Nature.

[45] Yang Q, Wang B, Zheng Q, Li H, Meng X, Zhou F, Zhang L (2023). A review of Gut Microbiota-Derived metabolites in Tumor Progression and Cancer Therapy. Adv Sci (Weinheim Baden-Wurttemberg Germany).

[46] Nagel G, Bjørge T, Jaensch A, Peter RS, Häggström C, Lang A, Engeland A, Teleka S, Jirström K, Lindquist D (2021). Metabolic factors and the risk of small intestine cancers: pooled study of 800 000 individuals in the metabolic syndrome and cancer project. Int J Cancer.

[47] Talmor-Barkan Y, Bar N, Shaul AA, Shahaf N, Godneva A, Bussi Y, Lotan-Pompan M, Weinberger A, Shechter A, Chezar-Azerrad C (2022). Metabolomic and microbiome profiling reveals personalized risk factors for coronary artery disease. Nat Med.

[48] Huang T, Long Y, Ou Y, Li J, Huang Y, Gao J (2023). Association between circulating fatty acid metabolites and asthma risk: a two-sample bidirectional mendelian randomization study. BMC Med Genom.

[49] López-Cotarelo P, González-Jiménez A, Agudo-Jiménez T, Abarca-Zabalía J, Aladro Y, Pilo B, Comabella M, Espino-Paisán L, Urcelay E (2021). Genetic variation in NDFIP1 modifies the metabolic patterns in immune cells of multiple sclerosis patients. Sci Rep.

[50] Xie W, Li J, Du H, Xia J (2023). Causal relationship between PCSK9 inhibitor and autoimmune diseases: a drug target mendelian randomization study. Arthritis Res Therapy.

