# Supplementary figures and images for: Integrated mendelian randomization analyses highlight AFF3 as a novel eQTL-mediated susceptibility gene in renal cancer and its potential mechanisms
Source: BMC Cancer. 2024 Jun 17;24:739. doi: 10.1186/s12885-024-12513-1 (PMC11181572; doi:10.1186/s12885-024-12513-1)

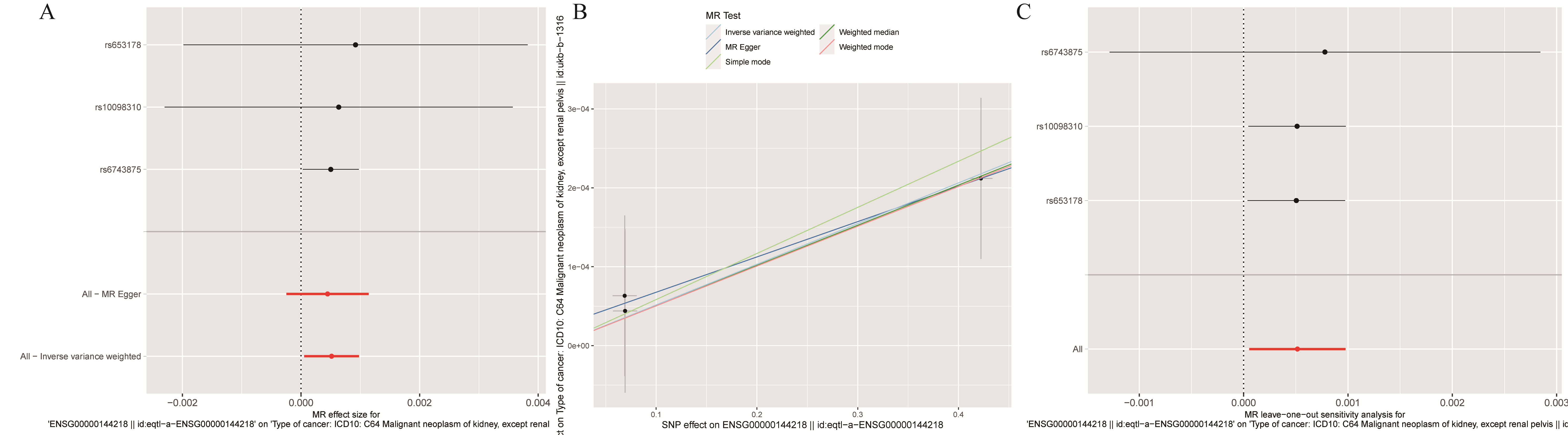

Supplement: Supplementary file 1 — Supplementary Material 1 [file 12885_2024_12513_MOESM1_ESM.png]

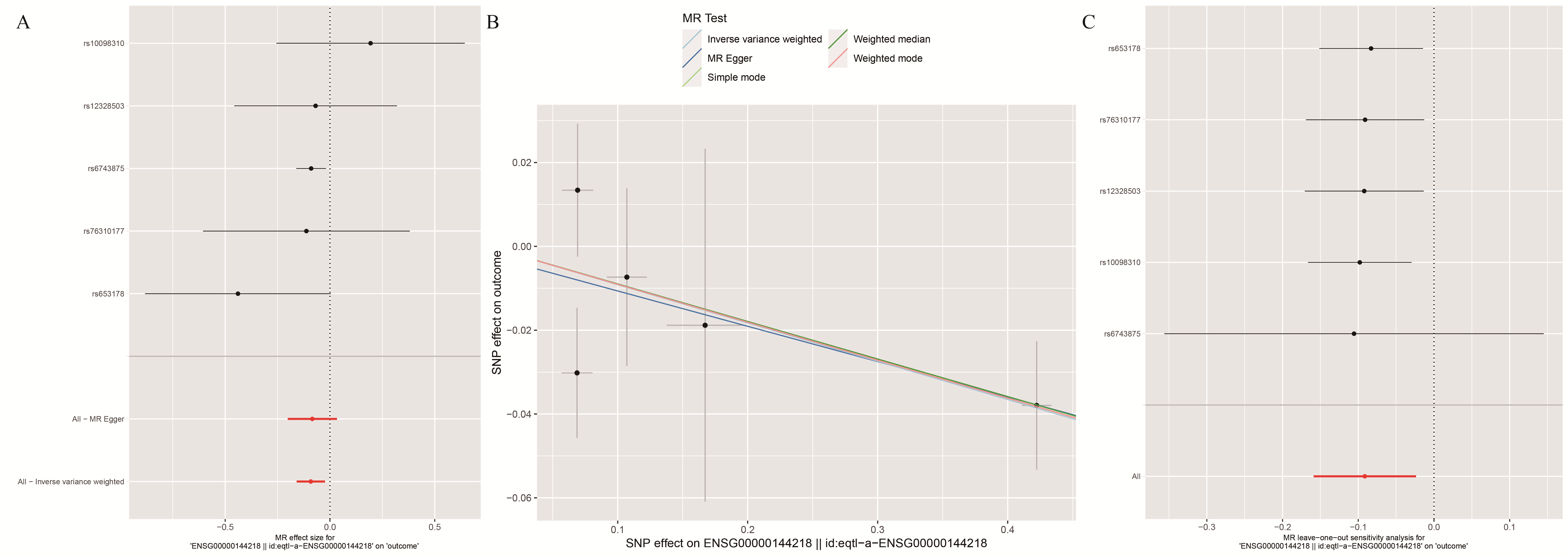

Supplement: Supplementary file 2 — Supplementary Material 2 [file 12885_2024_12513_MOESM2_ESM.png]

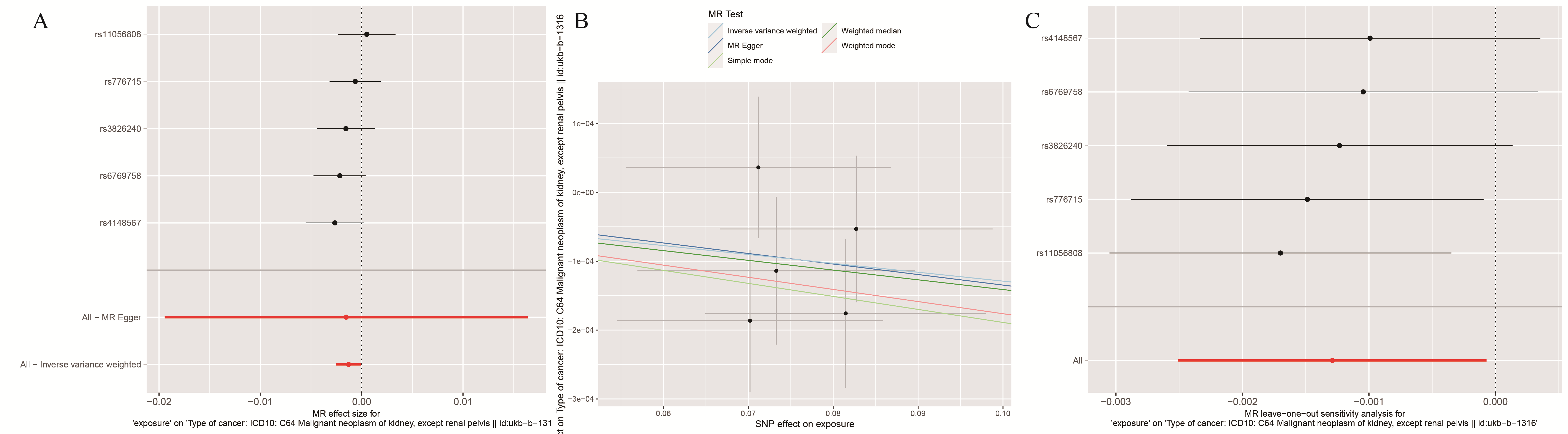

Supplement: Supplementary file 3 — Supplementary Material 3 [file 12885_2024_12513_MOESM3_ESM.png]
