# Supplementary material for: Integrated mendelian randomization analyses highlight AFF3 as a novel eQTL-mediated susceptibility gene in renal cancer and its potential mechanisms
Source: BMC Cancer. 2024 Jun 17;24:739. doi: 10.1186/s12885-024-12513-1 (PMC11181572; doi:10.1186/s12885-024-12513-1)
Supplement: Supplementary file 4 — Supplementary Material 4 [file 12885_2024_12513_MOESM4_ESM.docx]

**Supplement Materials**

**Figure S1**. Detailed (A) forest plot, (B) scatter plot, and (C) leave-one-out analysis for the causality of AFF3 eQTL susceptibility to RC;

**Figure S1**. Detailed (A) forest plot, (B) scatter plot, and (C) leave-one-out analysis for the causality of AFF3 eQTL susceptibility to the X-11315 metabolite levels;

**Figure S3**. Detailed (A) forest plot, (B) scatter plot, and (C) leave-one-out analysis for the causality of the X-11315 metabolite level susceptibility to RC;
